# Vibrational Stark Effects: Ionic Influence on Local Fields

**DOI:** 10.1021/acs.jpclett.2c01048

**Published:** 2022-05-27

**Authors:** Demelza Wright, Sara Sangtarash, Niclas S. Mueller, Qianqi Lin, Hatef Sadeghi, Jeremy J. Baumberg

**Affiliations:** † NanoPhotonics Centre, Department of Physics, Cavendish Laboratory, University of Cambridge, Cambridge CB3 0HE, U.K.; ‡ Device Modelling Group, School of Engineering, University of Warwick, Coventry CV4 7AL, U.K.

## Abstract

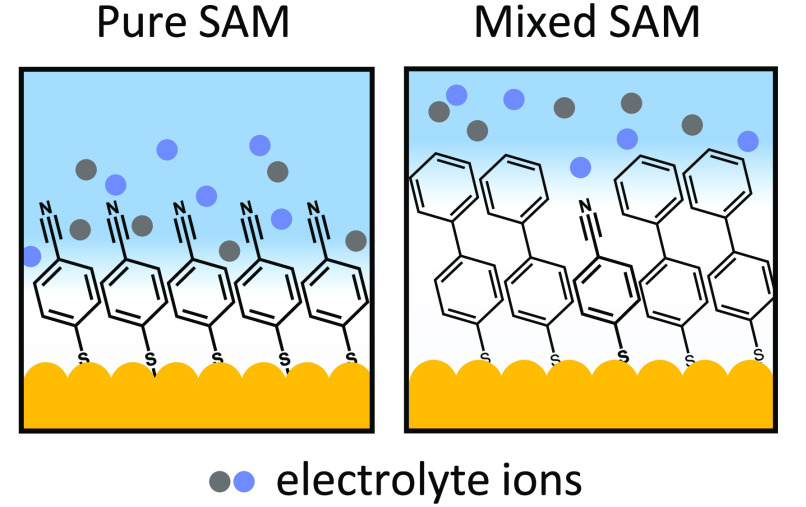

Molecules containing vibrational Stark shift reporters provide a useful tool for measuring DC electric fields *in situ*. To quantify this effect theoretically, density functional theory (DFT) calculations are usually utilized in a uniform electric field. However, using a combined theoretical and experimental study, we demonstrate here that uniform field DFT cannot simultaneously model the behavior of the three strongest vibrational modes in molecules forming a monolayer on an electrode. We show, by directly modeling ionic movement, that the measured Stark shifts are explained by partial electrical double-layer penetration into the molecular layer. This effect is sensitive to the local environment, and the Stark shifts can be fully suppressed experimentally by introducing a mixed molecular layer that prevents ionic double-layer penetration.

In vibrational spectroscopies such as Raman, infrared, and sum-frequency generation, spectral peak shifts can be observed in response to external static electric fields. Vibrational energy levels are modified in electric fields because of the vibrational Stark effect (VSE), which has been used to map fields near roughened electrode surfaces with surface-enhanced Raman spectroscopy (SERS).^1^ While SERS is valuable for studying interfacial chemical processes crucial for many applications, such as catalysis and energy harvesting,^2^ standard roughened gold (Au) electrodes complicate analysis because of their surface inhomogeneity and variable morphology. Here, the VSE is used to measure the electric fields on nanoparticle-on-mirror (NPoM) electrodes. Each NPoM is a highly reproducible bottom-up geometry that produces individual dimer-like nanocavities of optical volume <100 nm^3^ with Raman enhancements >10^8^, in which the mirror has a smooth, homogeneous surface.^3,4^ Early electrochemical work with similar geometries assigned bond shifts to Au–molecule bonding changes^5^ or to molecular flexing,^6^ not considering electrical double layer influences. Isocyanide groups, which are effective VSE reporters, shift in response to electric fields on NPoM electrodes^7^ but report only groups at the electrode/molecule interface, leaving the molecule/solution interface environment unexplored. Understanding ionic movement at both interfaces is crucial for surface-bound catalysis and molecular electronics.

To tackle this, we use a nitrile reporter and perform extensive density functional theory (DFT) calculations to explore the effect of a single ion pair on the VSE. We find that the application of a uniform field in DFT calculations is insufficient to describe our experiments despite the sensitivity of this model to molecular orientation, field direction, and field magnitude. We demonstrate instead the effectiveness of a simple ionic model that considers the effect of local electric fields induced by a single ion. Only including this allows DFT simulations to capture the spectroscopic features of key vibrational modes and to provide a physical depiction of the electrical double layer.

Nitriles are well-known Stark reporters that exhibit strong VSE shifts in response to electric fields,^8,9^ hence our choice of 4-mercaptobenzonitrile (MBN) here. Combined spectroscopic and electrochemical measurements are performed using a custom-built spectro-electrochemical cell (Figure 1a). Both NPoM and roughened Au electrodes are compared to explore the influence of nanoscale morphology on voltammetry, where the NPoMs form a precisely determined architecture in comparison to the inhomogeneous rough surface of crevices. Briefly, NPoMs are made by forming a self-assembled monolayer (SAM) on smooth gold before dispersing Au nanoparticles (NPs) on top. The result is a flat electrode appearing black in dark-field images with many individual bright scattering rings corresponding to NP positions (Figure 1b). By contrast, Au electrodes are roughened by the standard oxidation and reduction cycling (ORC) method in KCl electrolyte, and SAMs are directly formed onto the rough surface.^10^ Roughened Au electrodes have many scattering features in dark field images that correspond to a wide variety of Au structures on the surface (Figure 1c).

**Figure 1:**
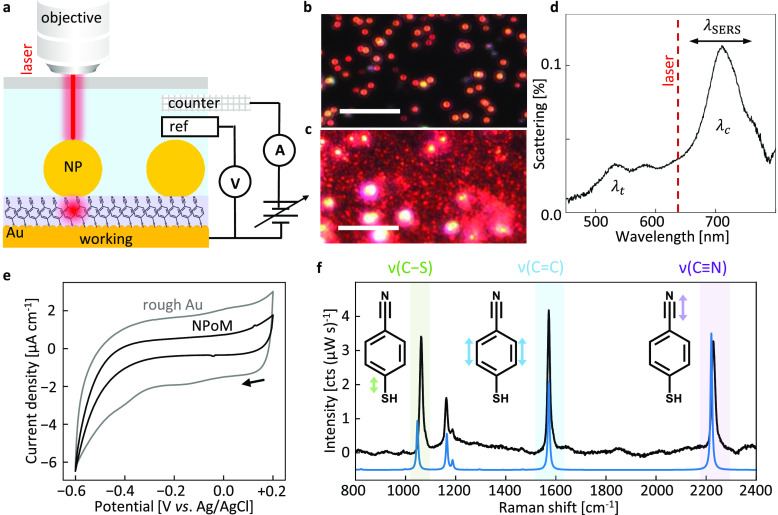
Spectro-electrochemical experiments. (a) Cell shown for NPoM, with contacted mirror forming the working electrode while the reference electrode (3 M Ag/AgCl) and counter electrode (Pt mesh) are immersed in solution. A 0.9 NA objective focuses light onto the sample and collects SERS through the coverslip. (b and c) Dark-field images of (b) NPoM and (c) roughened Au substrates (scale bars, 10 μm). (d) Typical dark-field scattering spectrum for NPoM with MBN monolayer and 60 nm AuNPs. Transverse () and coupled () plasmon modes are labeled; 633 nm laser (dashed line) is used for SERS. (e) Cyclic voltammograms for roughened Au (gray) and NPoM (black) substrates with MBN monolayers. Arrow indicates scan start; scan rate, 100 mV s^–1^; performed in N_2_-saturated 0.1 M KCl electrolyte. (f) SERS spectrum of MBN NPoM (black), with DFT spectrum for MBN (blue, scaling factor 0.965). Highlighted modes identified by DFT correspond to local MBN bond vibrations indicated by arrows.

Hundreds of NPoM junctions are characterized by automated dark field spectroscopy (Figures 1d and S1).^11^ Representative spectra show the expected NPoM scattering response, including transverse and coupled modes. Modeling the coupled mode resonance^12^ gives estimates of gap refractive index *n*_MBN_ = 1.4 and *d*_MBN_ = 0.9 nm, consistent with a near-upright aromatic SAM of DFT-predicted MBN length 8.3 Å.

Cyclic voltammetry measurements of MBN monolayers between +0.2 V and −0.6 V vs Ag/AgCl show a mostly capacitive response with the onset of reductive thiol desorption at potentials below −0.4 V (Figure 1e), albeit with very low current density (μA cm^–1^). Increased capacitive current on roughened Au is likely due to the larger surface area of the roughened electrode. The capacitive region remains identical before and after NP deposition (Figure S2), while the reductive wave decreases slightly. The SERS spectrum (Figure 1f, black) of MBN is characterized by three intense peaks assigned by DFT as ν(C–S), ν(C=C), and ν(C≡N) bonds (insets in Figure 1f). The DFT-calculated Raman spectrum (Figure 1f, blue) reproduces these features well.

VSE is a modulation of vibrational energy levels by an external electric field, and early electrochemical experiments utilized alkyl nitriles in an attempt to electrically decouple the nitrile group from the electrode.^13,14^ More recent studies do not make this distinction, and a range of molecules give little difference in Stark tuning rates unless the probe is covalently bound to the surface.^9,14−16^ Bond shifting can be caused by sources other than VSE, such as changes to bond character by electrochemical reduction or by direct electron injection from the electrode (analogous to electron-donating or electron-withdrawing groups).^17^ The MBN molecule can experience either direct reduction of the Au–thiol bond or electron donation to the nitrile group. To obtain a fuller picture of molecular changes with potential, we track all three characteristic Raman modes of MBN. We use a step-potential profile (Figures 2 and S3) between +0.2 V and −0.6 V vs Ag/AgCl during SERS measurements. As potential becomes more negative, both ν(C=C) and ν(C≡N) clearly redshift (2.5 and 5 cm^–1^, respectively) while ν(C–S) experiences a smaller (∼1 cm^–1^) blueshift. Over 100 spectra are recorded at each potential (including both repeated observations on a single NPoM junction and different NPoMs) to determine the variability of peak positions, with Lorentzian peak fit positions then averaged (Figures 2d–f and S4). Peak intensities increase at negative potentials, consistent with previous results that Raman cross sections increase in DC electric fields as electron redistribution modifies the molecular polarizability.^18^

**Figure 2:**
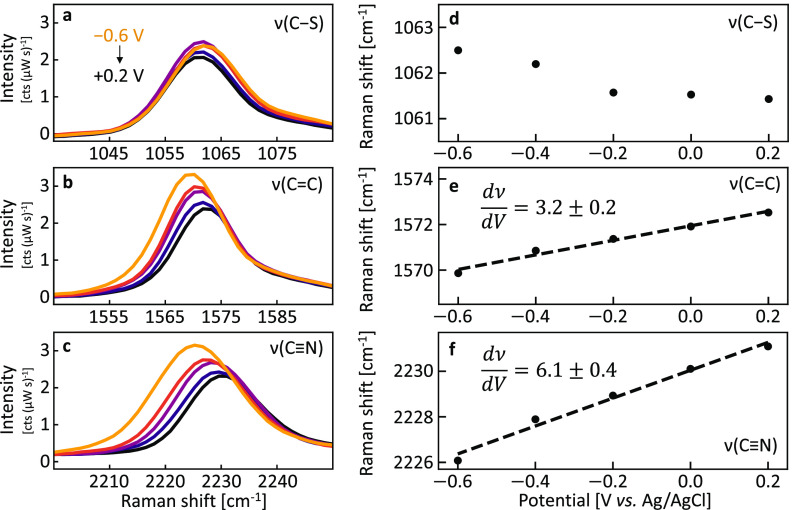
Potential-dependent SERS. (a–c) Averaged ( = 20) spectra of SERS peaks during chronoamperometry between 0.2 V and −0.6 V vs Ag/AgCl. Data is background subtracted and smoothed for clarity. (d–f) Averaged (=100) peak center positions from Lorentzian fits vs potential, for 0.1 M KCl electrolyte. Labels in (e) and (f) give Stark tuning rates in cm^−1^/V.

While ν(C=C) and ν(C≡N) exhibit a linear potential response (Figures 2 and S5), ν(C–S) shifts only below −0.4 V where thiol reduction currents start (Figure 1e). In the reductive desorption process, ν(Au*–*S) weakens so ν(C–S) should strengthen, as seen. Because the nitrile group follows a different trend from the ν(C*–*S) peak, thiol desorption is unlikely to contribute strongly to nitrile shifts. In comparison, roughened Au (Figure S5, purple) gives large spot-to-spot ν(C*–*S) peak variability in spectral position when compared to much smaller NPoM-to-NPoM variation, as well as less defined shift onsets and less consistent reversibility, likely because of nanoscale inhomogeneities.

The gradients (Figure 2e,f) give the Stark tuning rates, which match those expected for the nitrile VSE.^15,19^ Similar values from roughened Au at supporting electrolyte concentrations >10 mM (Table S1) indicate that the DC field at the MBN/AuNP interface is identical to that at the MBN/solution interface. The tuning rate of ν(C≡N) decreases at lower electrolyte concentrations (Figure S5). The extent of the electrical double layer increases as electrolyte concentration decreases, approximated by the Debye length () as a function of solution permittivity and concentration.^20^ For KCl electrolyte 0.94 nm,  3.0 nm, and  9.5 nm. Using these values and the Gouy–Chapman–Stern model of the double layer (not accounting for SAM effects), it follows that a Stark probe will experience smaller values of electric field (potential gradient) as electrolyte concentration decreases (Figure S6).^1^

These effects cannot be accounted for by Ohmic potential drops. Despite the expected large cell resistance (∼1 kΩ at 100 mM KCl) in these cells, the low currents (<1 μA) give very small potential drop (<1 mV) which hence does not affect measurements appreciably (see SI Note 1 for further discussion).

The structure of electrical double layers on bare electrodes is still debated, with recent emphasis on specific ion effects.^21^ The matter is even less clear when surface adsorbed species are present on the electrode, and little consistency is to be found in the literature. While some researchers assume that ions do not penetrate the SAM layer,^22^ or indeed that the SAM acts as a dielectric,^23^ others have used a model where the double layer forms within the SAM relatively unimpeded.^14,17^

Our finding that the tuning rate decreases with electrolyte concentration implies that the electrical double layer at least penetrates the nitrile moiety. This is further supported by repeating experiments using roughened Au substrates (Figure S5), recovering similar trends despite increased spot-to-spot variability.

While nitrile VSE is well-documented, to our knowledge no previous works report experimental changes to ν(C=C) with potential.^6,15,19,24^ Previous authors working with MBN pass no comment on this mode, even when reporting changes to smaller spectral peaks such as ν(C–H).^6^ We observe a strong, linear ν(C=C) shift with both NPoM and roughened Au substrates. Strong VSE is not usually expected for bonds without a large permanent dipole, although the strong electronic delocalization in phenyl molecules may give this mode more dipolar character. To test this, DFT calculations (see Computational Methods for details) were performed in a simulated uniform DC electric field (Figure S7). While all three of ν(C*–*S), ν(C=C), and ν(C≡N) are predicted to shift in a DC field, those shifts occur in the wrong ratio (for example ν(C=C) > ν(C≡N)), or in opposing directions to experiment (closest to our data is ref (19)). In addition, intense new modes are predicted around 900 cm^–1^ that are not observed experimentally.

To understand this further, exhaustive systematic DFT calculations (see Computational Methods for details) are performed in the presence of ions to explore the impact of a nearby ion–counterion pair on the local charge distribution (Figure 3). The counterion (solid green) is placed at  + 0.27 nm to ensure overall charge neutrality (where its exact lateral position has minimal effect on our results).

**Figure 3:**
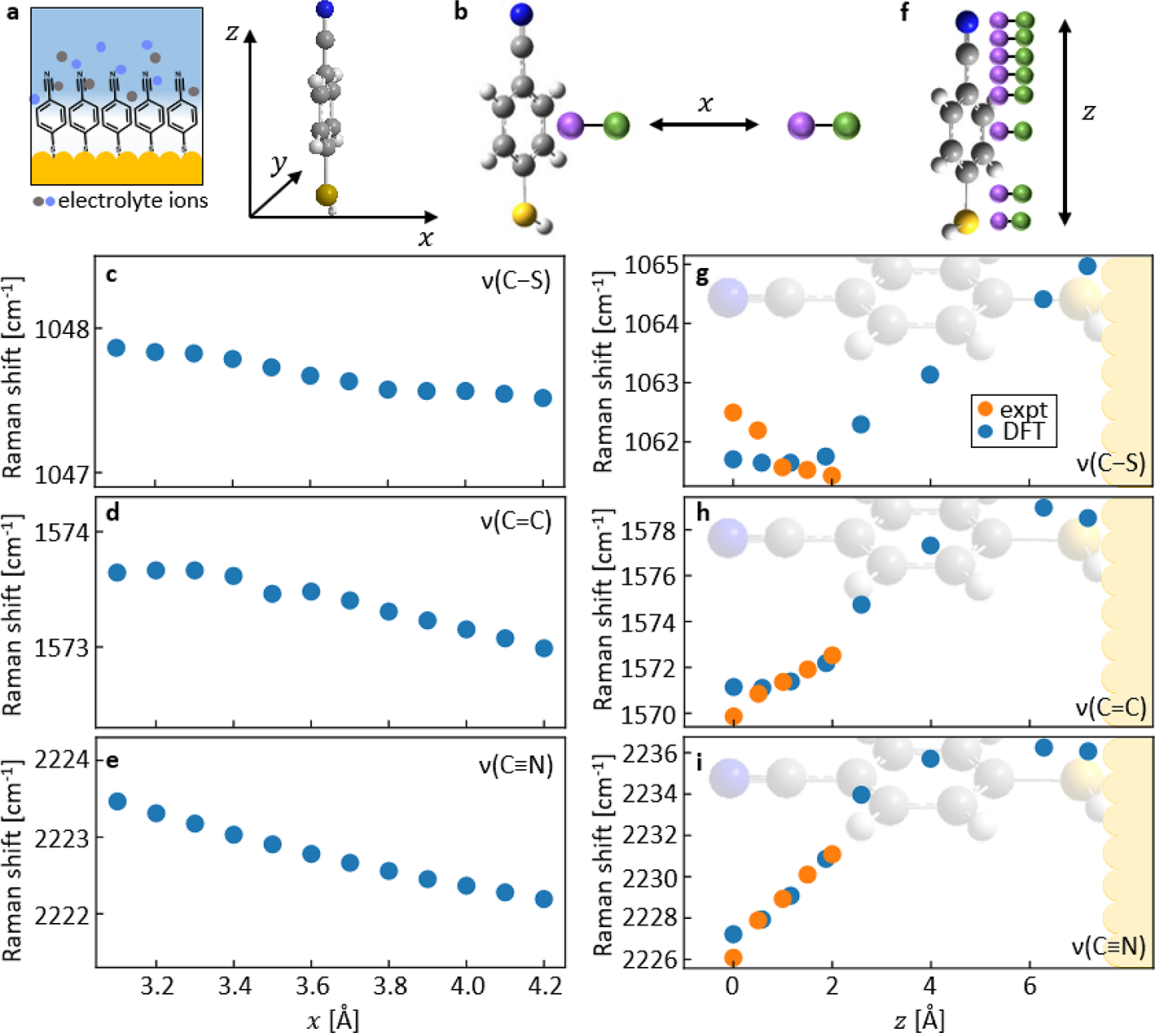
DFT-calculated Raman shifts with ion movement. (a) Schematic of MBN SAM and nearby ions, with axes for DFT calculations. (b) Schematic depicting lateral movement of the ion (solid purple) in the  direction, along with its counterion (solid green). (c–e) Calculated peak positions as ion and counterion move together along the -axis. (f) Schematic depicting vertical movement of ion and counterion along the -axis. (g–i) Calculated peak positions (blue) as ion pair moves along *z*, overlaid with experiment (orange). Experimental data rigidly offset by Raman shifts of (g) +15.3 cm^–1^, (h) +3.65 cm^–1^, and (i) +12.4 cm^–1^, scaled as *z*_expt_ [Å]= 2.5V[V] + 0.5, DFT scaling factor is 0.965.

The DFT model (depicted in Figure 3a) places the MBN upright along *z*, with the electrode in the *x−y* plane, and the phenyl rin*x*g at = 0 parallel to the *y−z* plane. The ion position is first varied along the *x*-axis (in all cases correspondingly also moving the counterion), approximating a variation in local ion concentration (Figure 3b), limiting the closest ion approach to be the sum of atomic and ionic radii.^25^ The vibrational peaks change as the ion moves closer to the molecule (Figure 3c–e) with charge distributions across MBN changing accordingly (Figure S8). Modifying the DC field while an ion is nearby (Figure S9) produces results similar to calculations with no ion (Figure S7). To match the observed shifts, this lateral model implies that Cl^–^ ions approach the molecule for negative electrode potentials, which is clearly unphysical (flipping the ion unit so that K^+^ is closest gives a similar problem, Figure S10). Lateral ion movement is thus not plausible to explain our observations.

We therefore take a different approach in our simulations. All double-layer models agree that ions migrate toward the electrode as the electrical double layer forms, which here is modeled as an ion moving along the -axis besides the MBN (Figure 3f). Our calculations show that all three peaks shift as the ion (K^+^ as shown in Figure 3f–i) moves past the nitrile group and approaches the center of the ring region. Comparing to experiment (Figure 3g–i, orange, using ), this model captures shifts in ν(C=C) and ν(C≡N) peaks in the correct ratio as the ion moves past the nitrile group toward the phenyl group. Apart from the thiol desorption effects discussed above, the ν(C–S) is correctly predicted to shift only a little. Interpreted literally, these results imply that at these potentials, ions are restricted from approaching the bottom electrode any more closely than the phenyl ring group and that the presence of a single electrolyte ion per molecule is sufficient to induce the Stark shifts observed.

Although this simple model does not consider interactions such as molecule–molecule, ionic screening, long-range interactions, or ionic hydration, good quantitative agreement is found between calculations and experiment. These effects can be modeled by the selected  value for the average ion pair position. This implies that the main contributing factor to Stark shifts in SAM layers can be better described by the local interactions of ions than by an average linear field. Physically, it is certainly feasible in a densely packed SAM that ions can closely approach the molecular headgroups and in so doing, modulate their vibrational energies. We emphasize that no other model can convincingly reproduce both ν(C=C) and ν(C≡N) shifts in the MBN molecule. Repeating these calculations now also including Au (Figure S11) gives no significant difference, showing that image charge effects are not dominant.

To demonstrate how the double-layer penetration can be controlled, two different molecules are now mixed within the SAM, combining MBN and 4-biphenylthiol (BPT), which modifies the ionic landscape around the MBN nitrile. Three MBN:BPT solution ratios are chosen when preparing the mixed SAMs: 100:0, 50:50, and 05:95, giving SERS spectra with the corresponding ratio of SERS intensities (Figure 4a). Because BPT is a longer molecule than MBN, its upper phenyl group should extend beyond the MBN nitrile (as depicted schematically in Figure 4b). Potential-dependent SERS measurements reveal a marked difference in ν(C≡N) Stark tuning rate with MBN:BPT ratio, decreasing from a maximum of 5.8 ± 0.2 cm^–1^/V in pure MBN to full suppression at 05:95 (Figure 4c). The MBN Stark tuning rate is thus very sensitive to the local ion concentration and shows that phenyl groups can form a barrier to ions penetrating inside a SAM.

**Figure 4:**
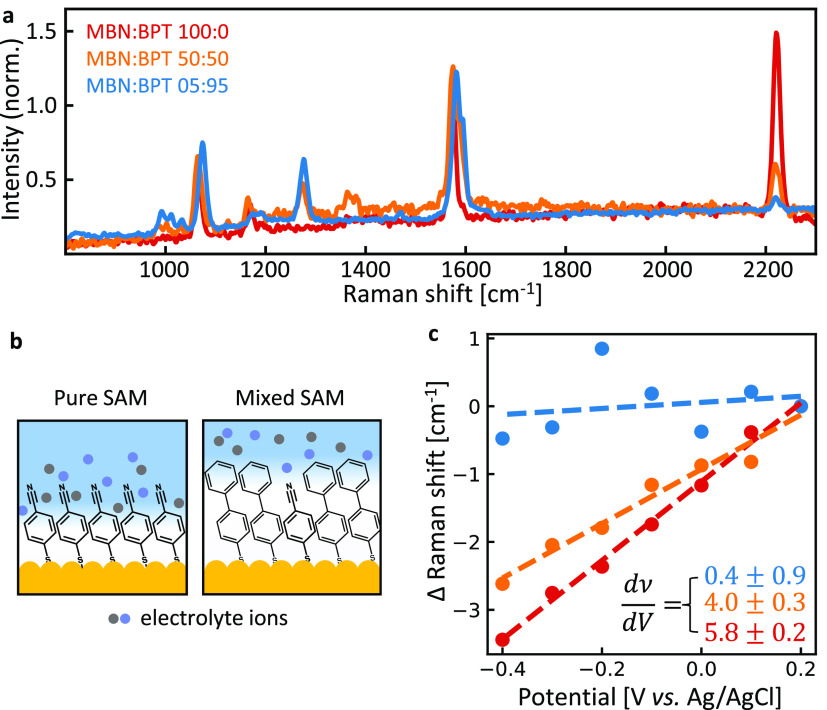
Mixed SAM experiments. (a) Representative SERS spectra on individual NPoMs for mixed MBN:BPT SAMs, normalized to BPT line at 1585 cm^–1^. Labels give molar mixing fraction. (b) Schematic of electrical double-layer ions in vicinity of pure and mixed MBN SAMs. (c) Stark shift data for ν(C≡N) mode in pure and mixed MBN SAMs. Colors correspond to spectra in panel a. Electrochemical conditions match those in Figure 2. Tuning rates given in cm^–1^/V.

Taken together, the combined DFT and experimental analysis of electrochemical Stark effects show that the vertical movement of ions by ∼2 Å can account for substantial vibrational mode shifts throughout the whole molecule. Further, the presence of another molecule at even a 50:50 ratio can suppress this effect by modifying the local ionic environment. We thus emphasize that caution must be taken when extracting quantitative information about the local environment on electrode surfaces from data on a single reporter bond using linear field models. As we show, much more detailed models for the SAM–electrolyte interaction must be considered. When modeled with care, the VSE can be a successful tool for gauging even complicated heterogeneous electric fields in biological systems.^16,26,27^ Of particular interest will be future explorations of the ionic penetration of organic electrolytes and ionic liquids into SAMs, about which much less is currently known.

In summary, we studied very reproducible Stark shifts in MBN monolayers and observed shifting of multiple vibrational modes that are not well-captured by linear field models. By modeling the influence of an electrolyte ion pair in close proximity to each molecule, we are able to reproduce the experimental results and predict the localization of ions in the molecular vicinity to <1 Å. Using mixed SAMs modifies the local ionic distribution and prevents ions from penetrating the SAM. This has important consequences for driving-force-sensitive surface reactions taking place in mixed, multimolecular, or “dirty” environments.

## Experimental Methods

All chemicals were purchased from Sigma-Aldrich, unless stated otherwise, at the highest purity available and used as received.

Au electrodes were fabricated by thermal evaporation. A 5 nm layer of Cr was evaporated onto a rotating silicon wafer, followed by a 50–100 nm layer of Au. Silicon wafers were then diced and cleaned by rinsing with EtOH and drying in N_2_. To make NPoM samples, SAMs were formed by immersing these chips in 1 mM MBN in EtOH for 22 h, then rinsing with EtOH, and drying in N_2_. AuNPs were dispersed onto the SAM by mixing 1 mM NaCl with 60 nm citrate buffered AuNPs (BBI Solutions) in 1:6 v:v, then drop casting 40 μL onto the SAM surface for 10 s before rinsing liberally with distilled water and drying in N_2_. Mixed SAM layers were made by mixing 50:50 and 05:95 v:v MBN and BPT solutions of 1 mM and proceeding as above.

Electrochemically roughened gold electrodes were made using an established oxidation and reduction cycling method.^10^ Briefly, Au films (before SAM deposition) were immersed in 0.1 M KCl and held at −0.6 V versus Ag/AgCl for 10 s, then swept to 1.1 V and held for 2 s. The samples were swept back to −0.6 V, and these steps were repeated 25 times. A SAM layer was then formed using the same parameters as above.

A 3D-printed 3-electrode cell was used for all spectro-electrochemical measurements. Au (NPoM or roughened Au) samples were used as the working electrode, a platinum mesh (Alfa Aesar) as counter electrode, and Ag/AgCl (3 M KCl, eDAQ ET072, Green Leaf Scientific) as reference electrode. The cell was closed by a 25  ×  25 ×  0.2 mm^3^ glass coverslip. Sample-to-coverslip distance is approximately 0.3 mm, to allow high-numerical-aperture (NA) collection of SERS scattering. Electrochemical measurements were recorded on an Autolab PGSTAT204 (Metrohm).

Measurements were recorded with a modified Olympus BX51 microscope coupled to a 633 nm laser and an incoherent white light source. Excitation and collection were performed through an Olympus 0.9 NA objective. SERS spectra were recorded by an Andor CCD camera coupled to a Triax 320 spectrometer. Dark-field scattering spectroscopy was collected by a fiber-coupled OceanOptics QE65000. Automated scans were performed using a Python particle-tracking code.^11^ A standard diffuser was used as a reference to normalize white light scattering.

## Computational Methods

The ground-state geometries of MBN in the absence and presence of ions and gold electrodes were obtained after performing geometry optimization using the Gaussian g16^28^ implementation of density functional theory. B3LYP hybrid functionals with Def2QZVP basis set and tight convergence criteria were used with a quadratically convergent SCF procedure. We then calculate the Raman spectra as a function of the combination of the following variables: (1) external electric field  with different strength (0, 0.4, 0.8, 1.6 eV) parallel to the nitrogen–sulfur direction added to the calculation using electric multipoles implemented in Gaussian g16, (2) ion–counterion pairs with K and/or Cl facing the MBN, (3) presence or absence of gold electrodes, and (4) different positions of ion pairs relative to MBN. These results are discussed in the main text and Supporting Information.

## Supporting Information Available

Dark-field scattering spectra, cyclic voltammetry, unprocessed surface enhanced Raman spectra, Stark shifting data under different electrolyte conditions (NPoM and rough Au electrodes), discussion of cell resistance, and additional DFT spectra of MBN (under electric fields, with and without Au, and with different ion-pair orientation) (PDF)

## Notes

Source data can be found at DOI: 10.17863/CAM.84952.
