# Supplementary material for: Vibrational Stark Effects: Ionic Influence on Local Fields
Source: J Phys Chem Lett. 2022 May 27;13(22):4905–11. doi: 10.1021/acs.jpclett.2c01048 (PMC9189927; doi:10.1021/acs.jpclett.2c01048)
Supplement: Supplementary file 1 — jz2c01048_si_001.pdf [file jz2c01048_si_001.pdf]

Supporting information for

Vibrational Stark Effects: Ionic Influence on Local Fields

Demelza Wright[1], Sara Sangtarash[2], Niclas S Mueller[1], Qianqi Lin[1], Hatef Sadeghi[2], Jeremy J Baumberg[1*]

[1] NanoPhotonics Centre, Department of Physics, Cavendish Laboratory, University of Cambridge, Cambridge, UK

[2] Device Modelling Group, School of Engineering, University of Warwick, Coventry, UK

* j.j.baumberg@phy.cam.ac.uk

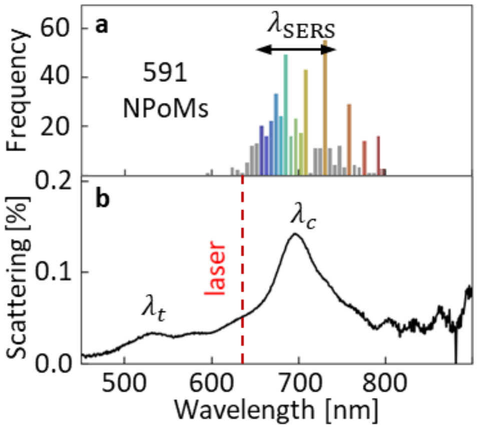

Figure S1 | Dark-field scattering. **a**, Histogram of NPoM coupled mode positions for 591 NPoMs with an MBN monolayer. **b**, Exemplar dark field scattering spectrum for an NPoM with MBN monolayer. Transverse (λ_t) and coupled (λ_c) plasmonic modes are labelled. Laser wavelength used for SERS λ_l=633 nm is labelled.

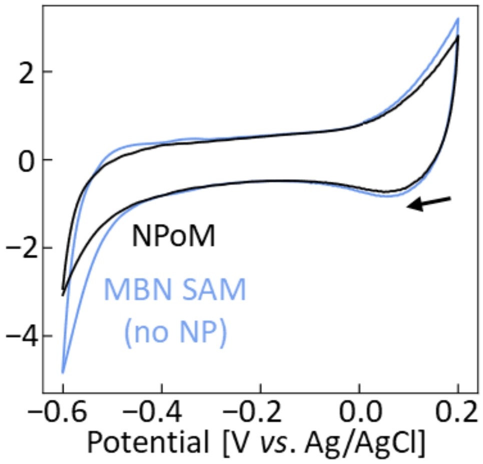

Figure S2 | Cyclic Voltammetry. Cyclic voltammograms for MBN monolayers before (blue) and after (black) NP deposition. Arrow indicates scan start. Scan rate = 100 mV s^{-1}, measurements performed in N_2-saturated 0.1 M KCl electrolyte at pH=7.

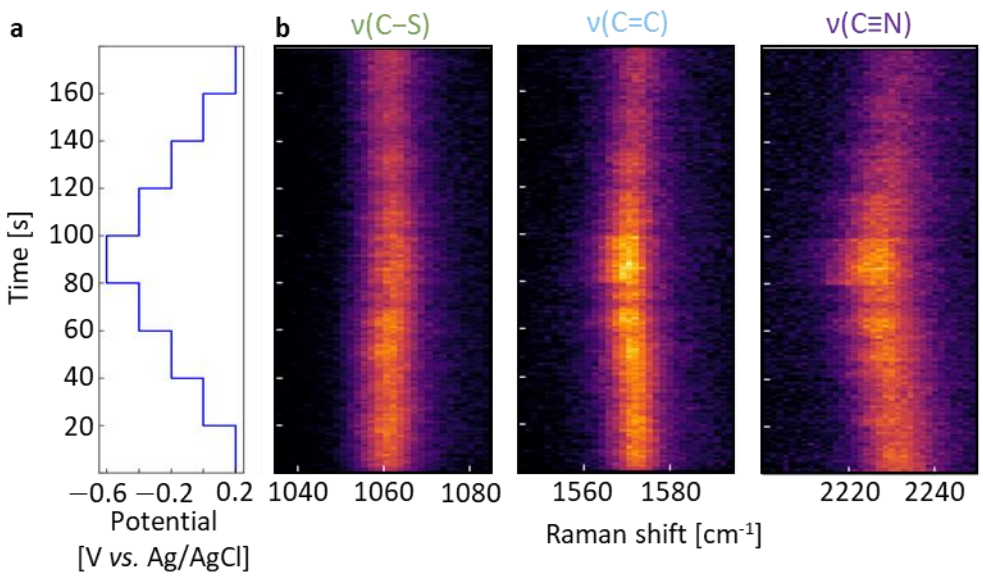

Figure S3 | Potential-dependent SERS. a, Stepped potential profile used for all experiments. **b**, Raw SERS data acquired under potential control. Data shown is for a NPoM sample in N_2-saturated 0.1 M KCl electrolyte. Excitation laser wavelength is 633 nm with a typical acquisition time of 1 s per spectrum, 100 µW incident power.

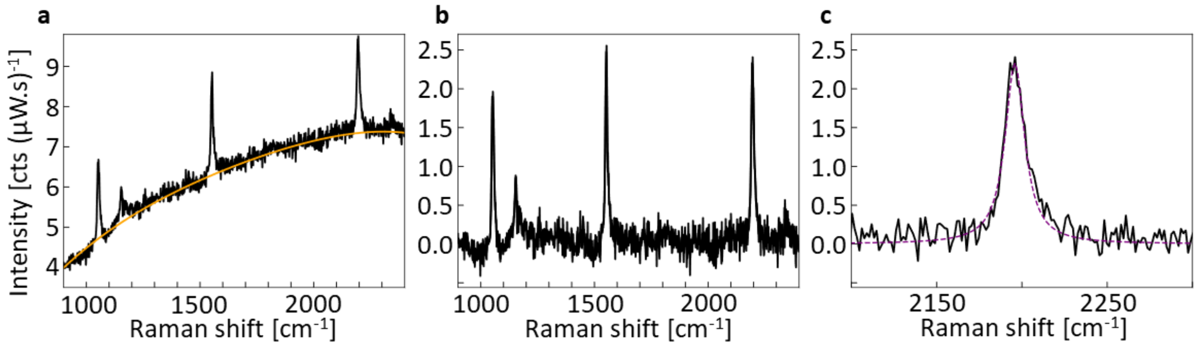

Figure S4 | Data analysis procedure. a, Example raw SERS spectrum with orange line indicating polynomial fit to background. **b**, Example of a background subtracted spectrum. **c**, Example of Lorentzian fit (purple dashed line) to data (black).

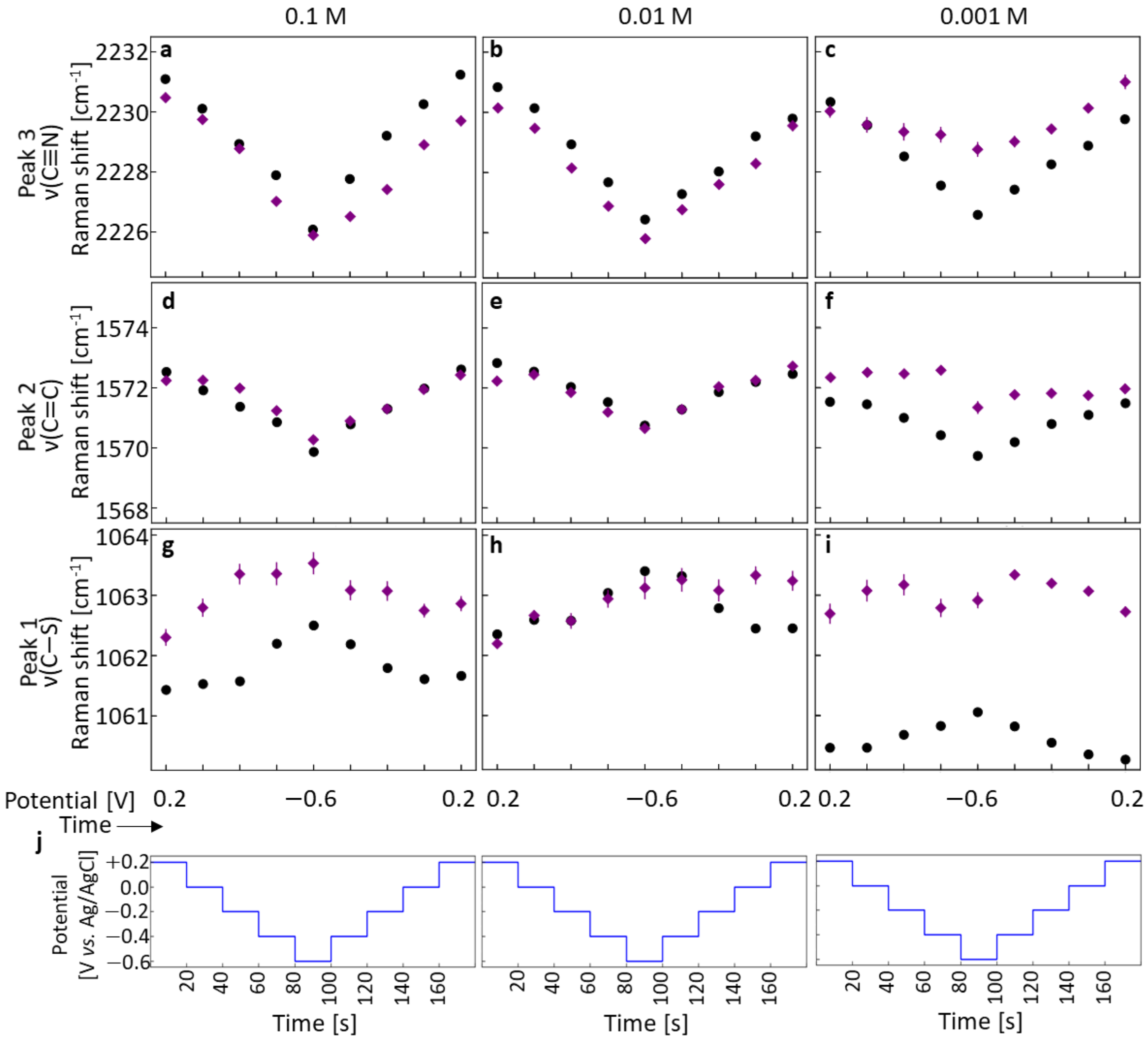

Figure S5 | Peak shift under potential control. a-i, NPoM (black circles) and rough Au (purple diamond) averaged (n=100) peak centre positions from Lorentzian fit *vs.* applied potential. **a-c**, ν(C≡N). **d-f**, ν(C=C), **g-i**, ν(C–S). **a,d,g**, Data using 0.1 M KCl electrolyte. **b,e,h**, Data using 0.01 M KCl electrolyte. **c,f,I**, Data using 0.001 M KCl electrolyte. **j**, Stepped potential profile used for all experiments, for reference.

Table S1 | Tuning rates calculated as a linear fit to averaged peak position *vs*. potential. Error quoted is standard error of regression.

Tuning rate [cm^{-1} V^{-1}]	KCl concentration					
	0.1 M		0.01 M		0.001 M	
	NPoM	Rough	NPoM	Rough	NPoM	Rough
ν(C–S)	-1.4 ± 0.3	-1.5 ± 0.3	-1.3 ± 0.2	-1.1 ± 0.2	-0.8 ± 0.1	-0.1 ± 0.2
ν(C=C)	3.2 ± 0.2	2.5 ± 0.6	2.6 ± 0.2	2.2 ± 0.5	2.3 ± 0.3	1.0 ± 0.8
ν(C≡N)	6.1 ± 0.4	5.9 ± 0.5	5.6 ± 0.3	5.6 ± 0.3	4.8 ± 0.1	1.4 ± 0.2

SI Note 1

The Ohmic drop was measured in the spectroelectrochemical cell using an MBN NPoM sample in KCl electrolyte at 100 mM, 10 mM and 1 mM concentrations.

Two methods were used: i-Interrupt and positive feedback using the Nova 2.1.4 software and Autolab PGSTAT204.

Electrolyte concentration (mM)	i-Interrupt resistance (Ohm)	Positive feedback resistance (Ohm)
100	610	1100
10	1900	2400
1	5600	13000

Taking typical chronoamperometry measurements and using largest resistance for each concentration:

Electrolyte concentration (mM)		Applied potential (V)					Change dV
		0.2	0	-0.2	-0.4	-0.6	
100	Current (A)	3E-8	6E-8	7E-8	2E-7	1E-6	
	iR (mV)	0.03	0.07	0.08	0.2	1.1	-0.14%
10	Current (A)	4E-8	7E-8	9E-8	4E-7	2E-6	
	iR (mV)	0.1	0.2	0.2	1.0	4	-0.46%
1	Current (A)	4E-8	6E-8	7E-8	2E-7	7E-7	
	iR (mV)	0.5	0.7	0.9	2.2	8.7	-1.2%

Change dV refers to by what amount the interval +0.2V to -0.6 V (i.e. dV used for Stark tuning rate calculations) changes in light of Ohmic potential drop. In all cases, Stark tuning rate remains within error reported in Table S1.

In conclusion, the Ohmic potential drop does not influence our data strongly.

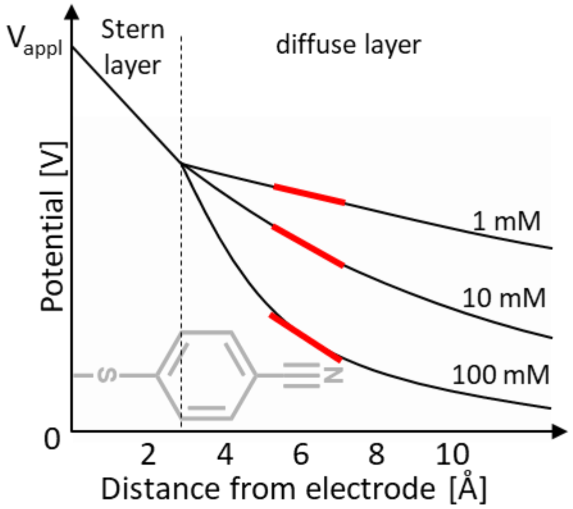

Figure S6 | Schematic representing a model of the electrical double later formed across a MBN SAM.

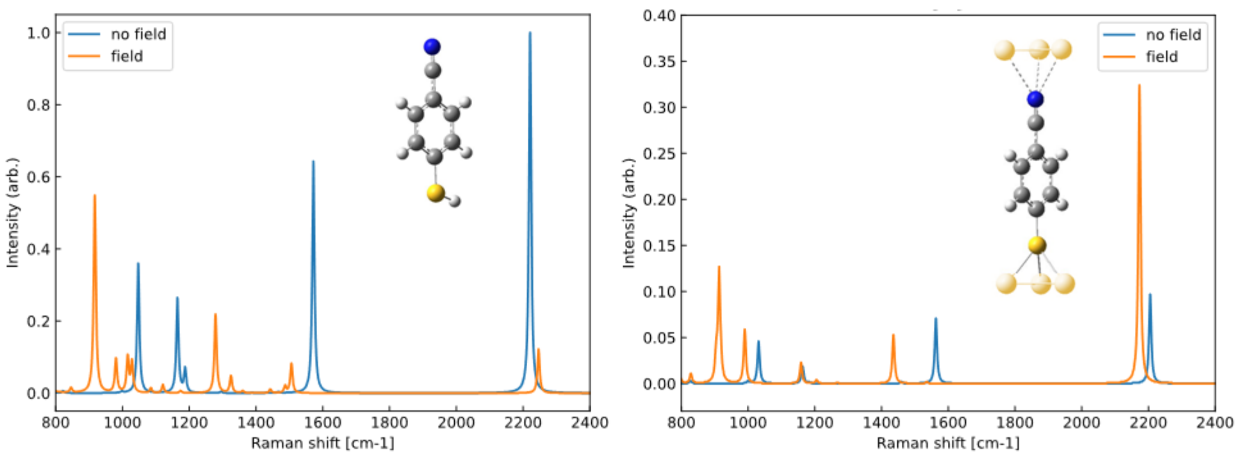

Figure S7| Influence of 1.6 eV DC electric field from sulphur (negative) to nitrogen (positive) on DFT-calculated MBN spectra.

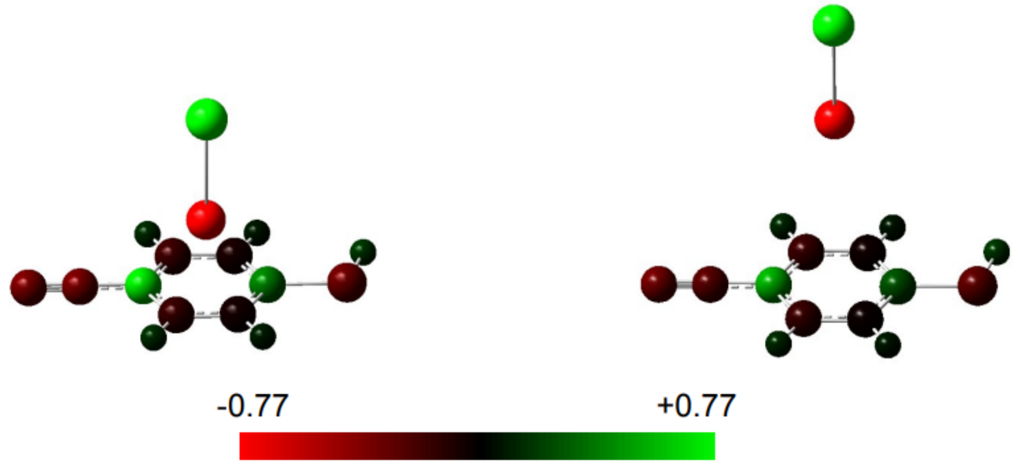

Figure S8 | Mulliken charge as ion approaches MBN.

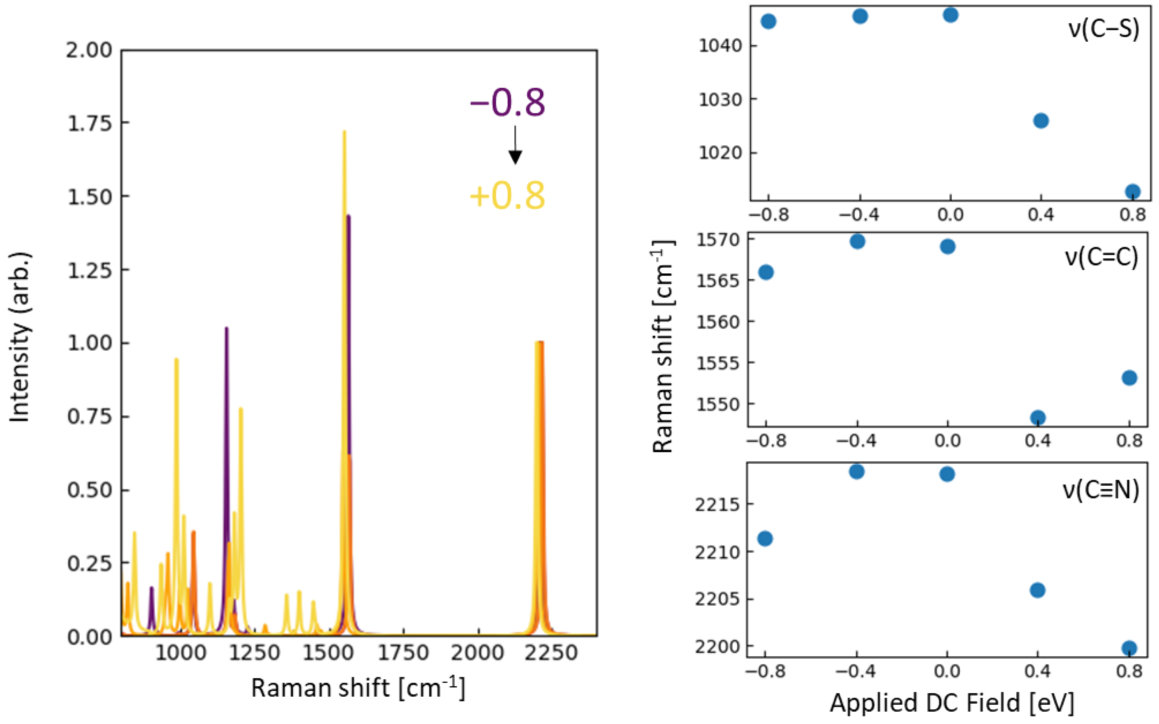

Figure S9 | Influence of DC electric fields on MBN spectra with nearby ion.

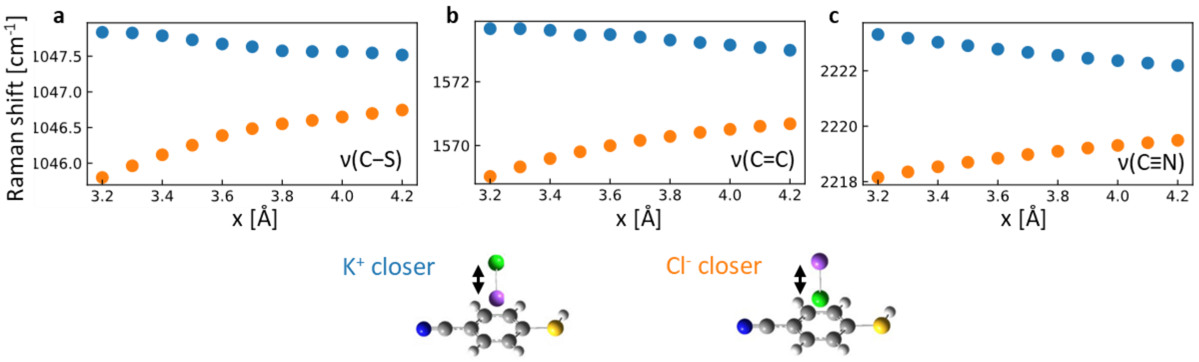

Figure S10 | DFT calculated peak positions *vs.* ionic position with K+ closest. a ν(C−S), **b**, ν(C=C), **c**, ν(C≡N).

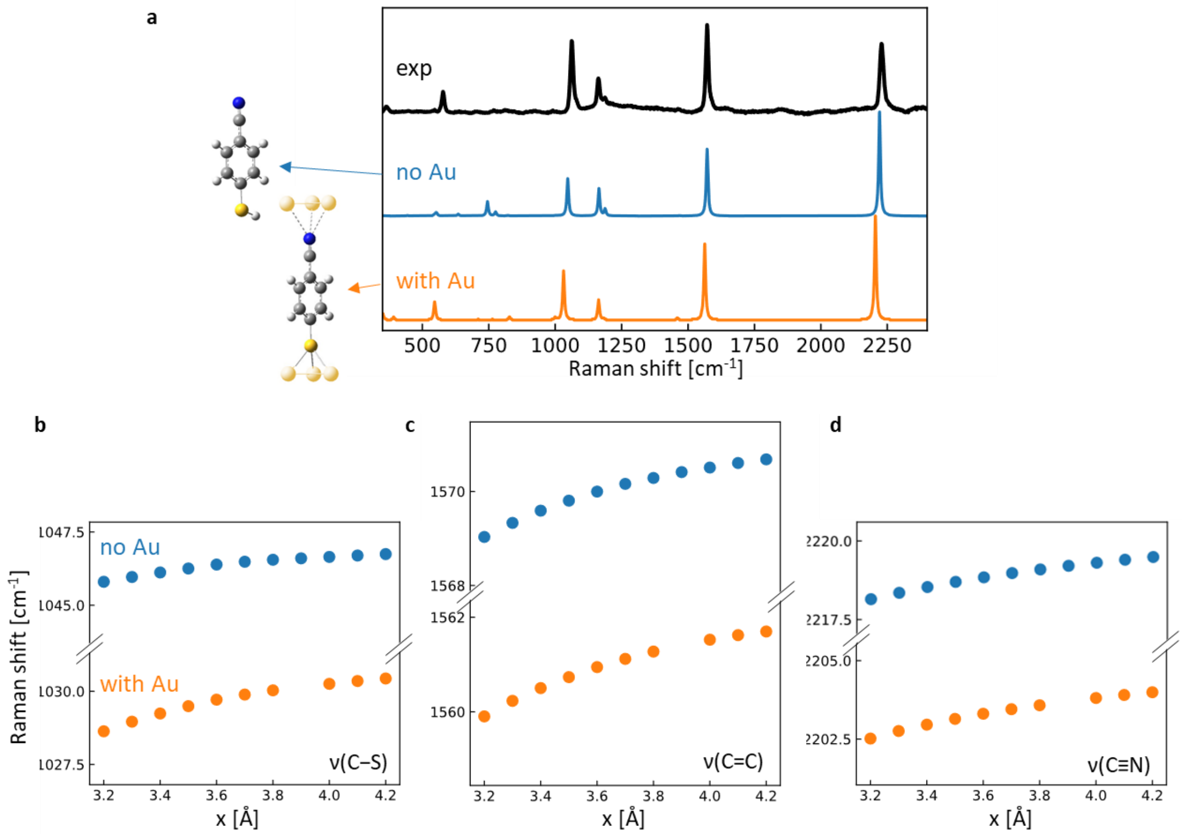

Figure S11 | Exploring the impact of Au on DFT-calculated Raman spectra. **a**, DFT-calculated spectra with and without Au, compared to experiment. **b-d**, Shifts of vibrational modes in response to an ion moving along the x-axis, with and without Au.
